# Double Orifice Mitral Valve and Bicuspid Aortic Valve: Pieces of the Same Single Puzzle?

**DOI:** 10.1155/2015/305142

**Published:** 2015-04-09

**Authors:** Faysal Şaylık, Ferit Onur Mutluer, Aydın Tosu, Murat Selçuk

**Affiliations:** ^1^Department of Cardiology, Van Region Training and Research Hospital, 65300 Van, Turkey; ^2^Kanuni Sultan Süleyman Training and Research Hospital, 34303 Istanbul, Turkey

## Abstract

Double orifice mitral valve is a very rare congenital abnormality. Well known associations of this pathology with other congenital lesions point to a complex and central pathophysiological mechanism leading to a sequence of pathologies. These associations have long been realized and arbitrarily defined as Shone complex. We would like to present a 21-year-old patient with double orifice mitral valve associated with bicuspid aortic valve, with a brief review of the literature on possible central mechanisms leading to different subsets of congenital abnormalities involving these two.

## 1. Introduction

Double orifice mitral valve is a very rare congenital abnormality [1]. The left atrium opens to the left ventricle via mitral valve orifice divided into two by a fibrous tissue. Isolated occurrence of this abnormality is an even rarer phenomenon. Well known associations of this pathology with other congenital lesions point to a complex and central pathophysiological mechanism leading to a sequence of pathologies. These associations have long been realized and arbitrarily defined as Shone complex. We would like to present a case with cooccurring double orifice mitral valve and bicuspid aortic valve, with the aim of drawing attention to possible underlying mechanisms.

## 2. Case Report

A 21-year-old man presented to our outpatient clinic with exercise-induced chest pain and palpitations. There was nothing remarkable in medical or family history. Questioning of the functional status revealed NYHA class III dyspnea. Vitals were normal. Levine 3/6 apical diastolic murmur was noted in auscultation. ECG, chest X-ray, and routine blood work yielded normal results.

**Figure 1:**
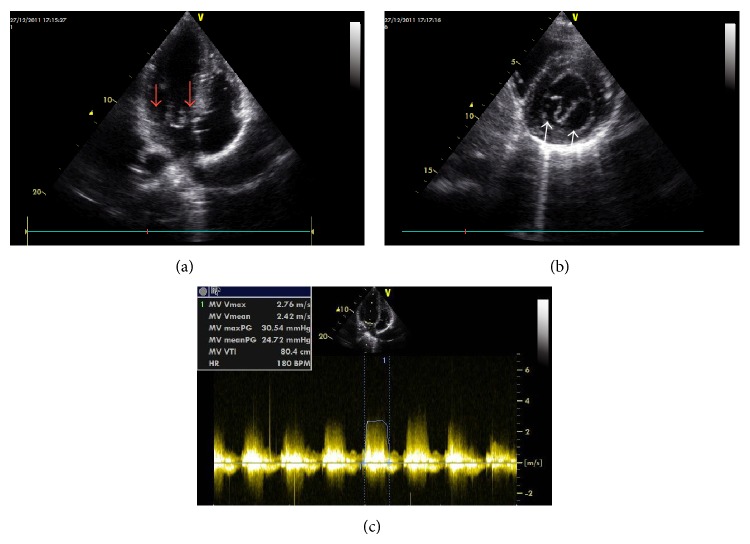
(a) Apical four-chamber view and (b) parasternal short axis view showing double orifice mitral valve. (c) Continuous wave Doppler tracings showing severe gradient over mitral valve.

**Figure 2:**
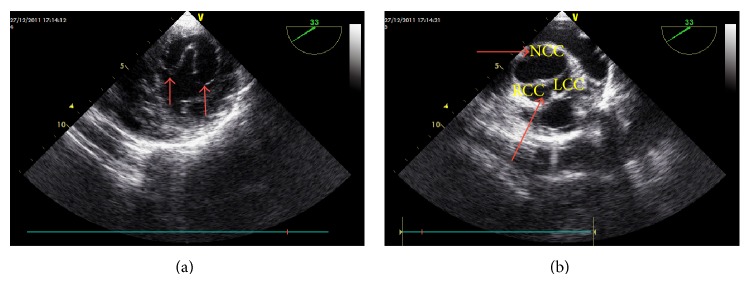
Modified section of mitral valve and short axis view of the bicuspid aortic valve with fusion of the left and right coronary cusps (LCC and RCC) in transesophageal echocardiography.

Transthoracic echocardiography clearly showed the mitral valve with 2 separate orifices. The gradients with continuous wave (CW) Doppler demonstrated severe mitral stenosis (24 mmHG mean gradient), in apical four-chamber and parasternal short axis (PSSX) view (Figures 1(a)-1(b)). An accompanying bicuspid aortic valve (BAV) with fusion of the left and right coronary cusps with insignificant gradient was observed in basal PSSX view (maximal and mean gradients of 18 and 12 mmHg, resp.). Transesophageal echocardiography (TOE) was performed to clarify mitral and aortic valve anatomy. DOMV was clearly visualized in TOE, and the gradient over the left ventricular outflow tract was localized to the level of the valve (Figures 2(a) and 2(b)). Coarctation of aorta or other associated congenital abnormalities was not detected. The patient was referred for surgery.

## 3. Discussion

DOMV is a very rare abnormality in which there are two separate orifices with separate respective chordal and papillary structures. This abnormality was first reported in 1876 by Greenfield [2]. Banerjee et al. reported incidence of this rare abnormality to be 0.05% [1]. DOMV is found to be associated with transposition of the great arteries, atresia of the left ventricle outflow tract, single coronary artery [3], ostium primum defect [4, 5], ostium secundum defect, ventricular septal defect and hypoplastic left heart syndrome [5], persistent left superior vena cava, complete endocardial cushion defect [6], sinus venosus type ASD, Ebstein's abnormality and right atrial mass [7], left ventricular noncompaction [8], and cardiac papillary fibroelastoma [9] and as part of various syndromes with extracardiac and cardiac manifestations [10]. Isolated DOMV is very rare [11].

Valve development is a complex process which is not completely understood. Biomechanical properties of the valve and the surrounding as well as signalling pathways regulating migration and differentiation of cellular components of precursor are thought to be responsible for the embryogenesis and pathogenesis of subsequent valvular diseases.

Certain genes implicated in pathogenesis of BAV are found to regulate overall differentiation and morphogenesis of all the valves. For example, NOTCH-1 signalling, which is implicated in pathogenesis of aortic valve disease, is shown to play important roles in normal valvulogenesis of all the valves by regulating activity of various other genes and transcription factors. Abnormalities in this gene or other associated genes and/or transcription factors might be responsible for the pathogenesis of DOMV and other associated valvular abnormalities, vascular diseases, and congenital heart defects.

## 4. Conclusion

DOMV is a very rare lesion which might be associated with various other left sided obstructive lesions. The hypothesis that a central genetic abnormality may result in DOMV and other associated abnormalities is attractive and should be tested. In the clinical settings, patients with DOMV should undergo cautious and detailed assessment for other components of Shone complex.

## Supplementary Material

Apical four-chamber view in transthoracic echocardiography showing opening of the double orifice mitral valve is seen in Supplementary Video 1. Video 2 demonstrates opening of the double orifice mitral valve in parasternal short axis view in transthoracic echocardiography. Video 3 shows opening of the bicuspid aortic valve in parasternal short axis view in transthoracic echocardiography.
